# Effects of a Low FODMAP Diet in Inflammatory Bowel Disease and Patient Experiences: A Mixed Methods Systematic Literature Review and Meta‐Analysis

**DOI:** 10.1111/jhn.70106

**Published:** 2025-08-05

**Authors:** Aubane Ville, Rebecca McRae, Jiayen Nomchong, Dianne P. Reidlinger, Alexandra R. Davidson, Heidi M. Staudacher, Loai Albarqouni

**Affiliations:** ^1^ Faculty of Health Sciences & Medicine Bond University Robina Queensland Australia; ^2^ Institute for Evidence‐Based Healthcare Robina Queensland Australia; ^3^ Food & Mood Centre, The Institute for Mental and Physical Health and Clinical Translation Deakin University Geelong Victoria Australia

**Keywords:** Crohn's disease, FODMAP, inflammatory bowel disease, irritable bowel syndrome, qualitative, RCT, ulcerative colitis

## Abstract

**Introduction:**

A low FODMAP diet reduces symptoms of irritable bowel syndrome (IBS), but its impact on inflammatory bowel disease (IBD) is less established. This systematic review aimed to: (1) assess the effect of a low FODMAP diet in IBD and (2) understand patient experiences when implementing the low FODMAP diet.

**Methods:**

Four databases (Medline, Embase, CINAHL and CENTRAL) were systematically searched. RCTs evaluating a low FODMAP diet in IBD on disease activity, inflammatory markers, gastrointestinal symptoms, and quality of life (QoL), and qualitative studies reporting patient experiences of low FODMAP interventions (in either IBS or IBD), were included. Outcome data were meta‐analysed as standardised mean differences or odds ratios. Qualitative data underwent content analysis using the Health Belief Model.

**Results:**

Five RCTs (*n* = 224) and two qualitative studies (*n* = 30 IBS patients, no studies in IBD) were included. Compared with controls, there was no effect of a low FODMAP diet on disease activity (Crohn's disease: SMD −0.33; −0.77, 0.11; ulcerative colitis: SMD −0.31; −0.78, 0.15) or faecal calprotectin (SMD −0.20; −0.49, 0.09), but lower severity of global IBS symptoms (SMD −0.56; −0.90, 0.23) and higher QoL scores (SMD 0.43; 0.05, 0.81) at end of intervention. Patients with IBS described implementation as burdensome (Severity, Barriers), inadequate professional support (Susceptibility) and found it difficult to interpret information (Susceptibility, Barriers). Meal plans and recipes (Cue to action and Self‐efficacy) gained through dietitian‐led information sessions were valued (Benefits).

**Conclusions:**

A low FODMAP diet does not impact IBD disease activity and inflammation markers, but leads to improved gastrointestinal symptoms compared with controls. The diet should be considered for improving functional gastrointestinal symptoms, but not an IBD treatment. There are minimal studies about patient experiences implementing the low FODMAP diet, all in IBS. Future research should assess patient experiences of low FODMAP diet implementation, specific to IBD.

**Protocol registration:**

PROSPERO CRD42023480762, Open Science Framework.

## Summary

- A low FODMAP diet reduces symptoms of irritable bowel syndrome (IBS), but its impact on inflammatory bowel disease (IBD) is unclear.
- These findings highlight the potential benefit of a low FODMAP diet for managing gastrointestinal symptoms and improving quality of life in patients with IBD experiencing IBS symptoms.
- Social challenges, the burden of implementation and the lack of tailored support were major barriers to following a low FODMAP diet in IBS, but there were no studies of patient experience in IBD.

## 1 Introduction

Inflammatory bowel disease (IBD), including Crohn's disease and ulcerative colitis, affects approximately 3.1 million people (1.3%) in the USA and 2.5–3 million people (0.4%) in Europe [1, 2, 3]. Beyond healthcare costs, IBD impacts activities of daily living, is associated with social stigma and impacts food‐related quality of life for patients [4, 5]. As the global burden of IBD continues to mount, there has been increasing interest in the influence of lifestyle and dietary factors in the development and treatment of IBD [4, 6].

Fermentable oligosaccharides, disaccharides, monosaccharides and polyols (FODMAPs) are osmotically active carbohydrates. Some of these are slowly or minimally absorbed in the small intestine (fructans, galacto‐oligosaccharides, polyols) and others are not absorbed in certain conditions (i.e., fructose when fructose intake exceeds GLUT5 capacity, lactose in lactase deficiency). Upon reaching the colon, the unabsorbed carbohydrates undergo rapid fermentation by bacteria, leading to the production of short‐chain fatty acids, carbon dioxide, hydrogen and methane. These gases are associated with gastrointestinal (GI) symptom provocation in individuals with irritable bowel syndrome (IBS) [7]. Evidence from a recent network meta‐analysis ranked the low FODMAP diet first against several other comparator diets for global symptoms, abdominal pain severity and bloating [8]. This dietary approach may also be relevant in IBD, considering over 30% of people with IBD will also experience IBS‐type symptoms [9].

Conventional IBD treatments target underlying inflammation and involve pharmacotherapy (e.g., aminosalicylates, corticosteroids, immunomodulators and biologics) and surgical intervention [10]. While research has predominantly focused on the impact of FODMAPs in IBS, there is also some evidence that FODMAPs exacerbate GI symptoms in individuals with IBD [11, 12], and manipulation of dietary FODMAPs is recommended in several global clinical guidelines for patients with IBD to treat functional symptoms [10, 13, 14, 15]. While there is promise for GI symptom relief with the low FODMAP diet in IBD, a robust synthesis has not yet been conducted. A recent meta‐analysis suggests clinical benefit of a low FODMAP diet in patients with IBD [16]; however, improper pooling of randomised controlled trials (RCTs) with observational studies critically limits its validity. Furthermore, a summary of the wider consequences such as the impact on gut microbiota and diet quality that have been identified in IBS has not yet been reported in IBD [17, 18, 19].

The challenges, potential risks and support needs of patients when implementing a treatment are critically important for long‐term adherence to an otherwise efficacious treatment. This is particularly so for the low FODMAP diet, because of the systematic phased approach involving restriction, reintroduction and personalisation. A dietitian is recommended for supporting patients through the diet programme, although not all patients have access or are referred for dietetic input. Adhering to the low FODMAP diet also poses potential impacts on gut microbiome [20], diet quality [18, 21] and social lives of patients and their families [17, 19]. Therefore, dietary assessment, counselling and monitoring from a dietitian is essential to optimising adherence to the low FODMAP diet, tailoring the diet to individual needs, and minimising its negative impacts [22, 23].

This systematic review aimed to synthesise high‐quality evidence on the potential impact of a low FODMAP diet on IBD disease activity, inflammatory markers, GI symptoms, quality of life, diet quality and the microbiome in adults living with IBD. Additionally, it aimed to evaluate the experiences and perspectives of individuals implementing a low FODMAP diet.

## 2 Methods

### 2.1 Design

We conducted a systematic literature review and meta‐analysis, with a qualitative synthesis of patient experiences. The review was guided by the methodology outlined in the Cochrane Handbook for Systematic Reviews and was reported in accordance with the Preferred Reporting Items for Systematic Reviews and Meta‐Analyses (PRISMA) statement [24, 25]. The review was registered on PROSPERO (CRD42023480762) and Open Science Framework.

### 2.2 Search Strategy

We searched four databases: Medline, Embase, CINAHL and Cochrane CENTRAL from inception to 17 October 2023, with no restrictions on language or date of publication. The search strategy (Supporting Information S1: 1) was designed in PubMed, using a combination of keywords and MeSH terms relating to IBD and FODMAP. The PubMed search string was translated to the other databases using Polyglot Search Translator [26], with the assistance of a specialist librarian. We also searched clinical trial registries (ClinicalTrials.gov and WHO ICTRP). Further, we conducted forward and backward citation analysis using SpiderCite on all included studies [27].

### 2.3 Eligibility Criteria

We included RCTs investigating the low FODMAP diet compared with a control group in adults (≥ 18 years) with a diagnosis of IBD (ulcerative colitis, Crohn's disease or unspecified IBD). Interventions that were implemented through counselling and/or controlled feeding strategies were included. Eligible control groups included groups that continued habitual diet or undertook a control or comparator diet (e.g., sham diet, high FODMAP diet). RCTs with one or more outcomes, including disease activity, inflammatory markers, global GI symptom severity, individual symptoms, quality of life, anthropometric data, dietary intake or microbiome, were included. Abstracts with no usable data were excluded. Mixed methods or qualitative studies that reported the experiences and perspectives of individuals who had implemented a low FODMAP diet were also included. We broadened the inclusion criteria for these studies to encompass individuals with IBS (for whom the low FODMAP diet was originally developed) as well as those with IBD. Considering the vast literature of the low FODMAP diet in patients with IBS, we also included this population, as both groups could guide health professionals to better support the implementation of a low FODMAP diet. Although there are likely to be differences in the experience of people with a diagnosis of IBD compared to IBS, it was considered that the experiences of people with IBS when undertaking this dietary intervention could be extrapolated to those with IBD. In particular, many of the facets of patient experience for people with IBS would be comparable to patients with IBD who have quiescent or mild inflammatory disease and whose GI symptom burden is functional in origin.

Three authors (A.V., R.M., J.N.) independently screened titles and abstracts of the search results in duplicate, against the prespecified inclusion criteria. Those not excluded at title and abstract stage were reviewed at full‐text stage in duplicate. Discrepancies were resolved through discussion among the authors until consensus was reached.

## 3 Data Extraction

Two authors (A.V. and R.M.) extracted data from included RCTs into a data extraction form developed a priori in Excel (Microsoft 365 MSO), and a third author (J.N.) independently cross‐checked the accuracy of the extracted data. Extracted data included author, country, year of publication, study design, baseline demographic, study duration, mode of delivery, nature of control diet, adherence to diets and outcomes. The outcomes included: (1) disease activity measured by validated tools; (2) inflammatory markers measured via faecal calprotectin and/or C‐reactive protein; (3) global GI symptom severity and individual symptoms (abdominal pain intensity and duration, abdominal bloating or distension severity, and flatulence) measured by validated questionnaires); (4) quality of life measured by a validated questionnaire; (5) dietary intake measured by a food record; and (6) microbiome composition measured using different techniques.

One author (R.M.) extracted data from the qualitative and mixed methods studies, including study title, author, publication year, location, healthcare setting, mean age, mode of education delivery, phenomenon of interest, analysis type and findings (themes); data were further checked by a second author (A.R.D.). Data reporting on experiences, enablers and barriers of implementing a low FODMAP diet in either IBS or IBD were extracted. Data included descriptions of patients' experiences, themes and categories developed by authors as well as participants' direct quotes.

## 4 Quality Assessment and Risk of Bias

For RCTs, two authors (A.V., J.N.) independently assessed the quality of included studies using the Cochrane Risk of Bias (RoB2) tool for disease activity, inflammatory markers, GI symptoms and quality of life outcomes [28].

Three researchers (A.V., R.M., J.N.), independently and in pairs, assessed the certainty of evidence for each outcome using the GRADE (Grading of Recommendations Assessment, Development and Evaluation) framework. Overall certainty of evidence was rated as high, moderate, low or very low. We described treatment effects using GRADE‐recommended language [29].

For qualitative and mixed methods studies, two authors (R.M., A.D.) independently applied the Mixed Methods Assessment Tool (MMAT) [30]. MMAT captured the appropriateness of qualitative and mixed methods for the research question, accuracy of data interpretation and extraction, data cohesion, sampling and analysis methods. Disagreements arising from the quality assessments were resolved by discussion or, when required, by consultation with a third author (L.A.).

## 5 Data Synthesis and Statistical Analysis

### 5.1 Quantitative—Meta‐Analysis

Meta‐analysis was performed by one researcher (A.V.) when outcomes were reported in two or more studies using consistent metrics and with a separate comparator group. The overall effect of interventions was calculated using the difference between end‐of‐trial values between groups. For individual symptoms, odds ratios (ORs) and change from baseline values were used. RevMan5 (Review Manager (RevMan) [Computer programme], Version 5.4.1, The Cochrane Collaboration, 2020) was used to conduct analyses and generate forest plots for each outcome with 95% confidence intervals (CIs). To account for heterogeneity between the studies, a random‐effects model was used to produce a pooled estimate of the mean difference (MD) or standardised mean difference (SMD). Statistical significance was set at *p* < 0.05. The degree of variance arising from between‐study heterogeneity was assessed and presented as *I* ^2^, with significant heterogeneity defined as *I* ^2^ ≥ 50%. SMD was used to calculate effect sizes where outcome data were reported in different units or where there was heterogeneity in outcome measures. Where sample size, median and interquartile range were reported, the estimated sample mean was calculated using online calculators applying the method from Wan and colleagues [31, 32]. To convert data reported graphically into numbers, the Web Plot Digitiser was used [33]. When exact *p* values were reported without CIs, that value was used alongside the MD to estimate both the standard error and standard deviations. SMDs were calculated for continuous outcomes and were converted into OR to homogenise data for meta‐analysis, employing the method proposed by Hasselblad and Hedges [34].

### 5.2 Qualitative—Content Analysis

Familiarisation with data from qualitative studies occurred by reading the research papers several times. Through this process, researchers (D.P.R., R.M., J.N.) extracted relevant content into Excel and identified potential themes to source an appropriate framework for summarising qualitative data. The Health Belief Model was chosen as it identifies enablers and barriers to health behaviour change [35], and therefore was ideal for identifying support needs. This model considers six domains and their relationship to behaviour change, including (1) perceived susceptibility, (2) perceived severity, (3) perceived benefits, (4) perceived barriers (5), cue to action and (6) self‐efficacy [35]. Data were inductively coded line by line by two authors (R.M., J.N.), guided by the selected framework and research question. Once coded, data were categorised, further refined with the input of the wider research team and then deductively placed into the Health Belief Model domains with exemplar quotes selected to demonstrate categories within each domain [35].

### 5.3 Change From Protocol

This paper now includes disease activity, rather than GI symptoms, as a primary outcome, as this was almost always reported in the RCTs as a key outcome together with GI symptoms.

## 6 Results

**Figure 1:**
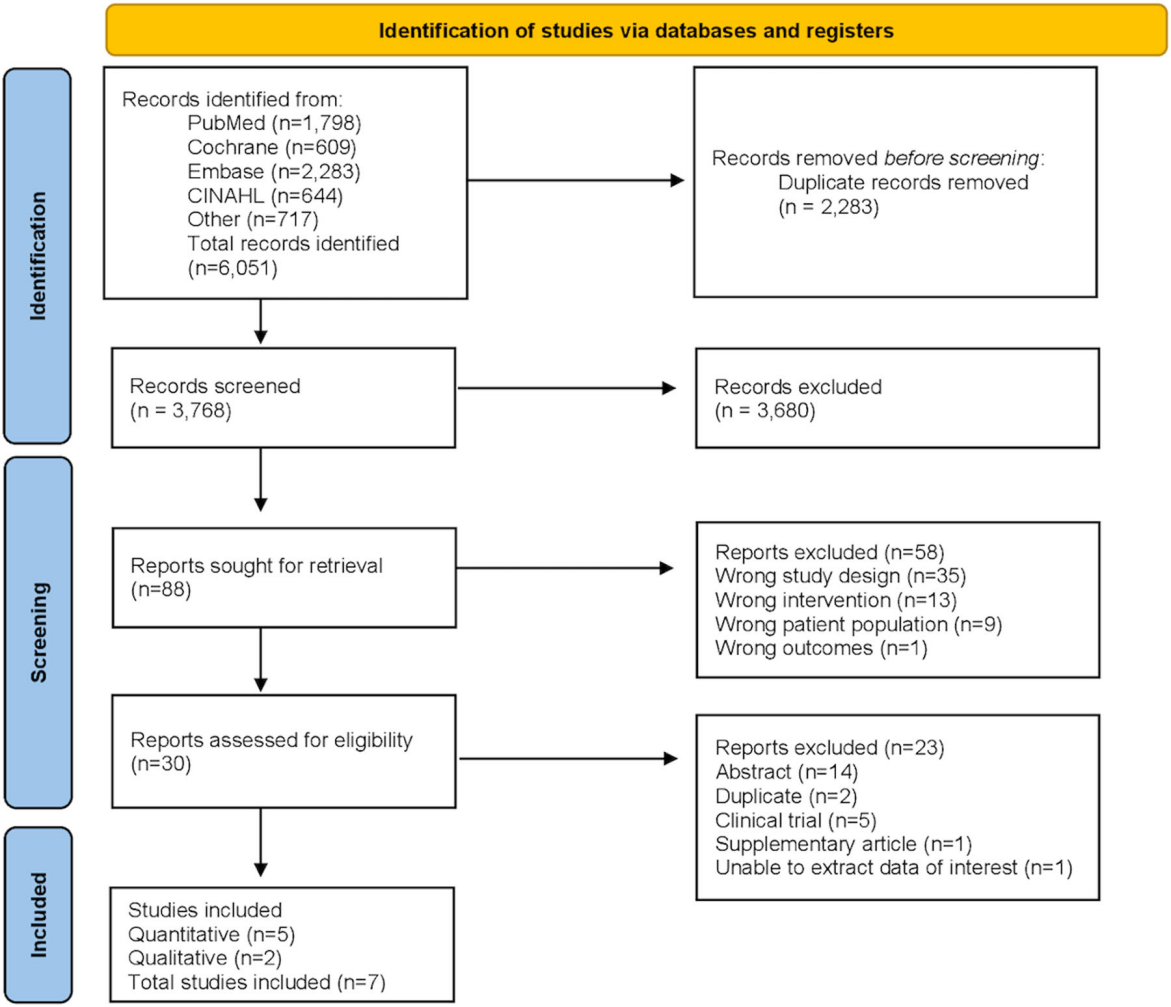
Prisma flow chart. From Page et al. [25].

We identified 6051 records, of which 3768 were unique after removal of duplicates. After title and abstract screening, we retrieved 88 full texts for eligibility. Overall, we included seven studies; five RCTs reporting the effects of a low FODMAP diet in individuals with IBD [36, 37, 38, 39, 40] and two studies with qualitative data in IBS [19, 41]; one of which was mixed methods [41] for which only the qualitative data were extracted (Figure 1). There were no studies of patient experience of implementing the diet in IBD. From the original search, we identified one further potentially eligible qualitative study [42]; however, despite contacting the authors, we were unable to retrieve the full text.

### 6.1 Characteristics of Included Studies

There were five RCTs (*n* = 224) included with intervention duration ranging from 2 to 9 weeks (Table 1). These included three trials of patients with Crohn's disease and patients with ulcerative colitis [36, 37, 40], one trial of patients with Crohn's disease [38] and one trial of patients with ulcerative colitis [39]. One trial included patients either in remission or with mild to moderate disease activity [40], one trial included patients in remission or with mild disease activity [36], and three trials included patients in remission only [37, 38, 39]. In each of the included trials, there was no significant difference in baseline symptom severity between the intervention and control groups [36, 37, 38, 39, 40].

**Table 1:** Characteristics of included randomised controlled trials of a FODMAP diet in IBD.

Country, Design	Population Total *n* (% female) Disease type (*n*)	Intervention duration (weeks)	Mode of delivery of the low FODMAP diet	Control	% Participants with high adherence to low FODMAP diet
Denmark, RCT, parallel [40]	*n* = 89 (75%) Patients with CD (*n* = 28) or UC (*n* = 61) in remission or with mild‐to‐moderate disease activity and coexisting IBS‐like symptoms	6	Dietary advice from a dietitian during a 1 h appointment and additional food lists, recipes and meal plans provided	Habitual diet	Not reported
UK, RCT, parallel [37]	*n* = 52 (56%) Patients with CD (*n* = 26) or UC (*n* = 26) with GI symptoms	4	Dietary advice from a dietitian during a 20 min appointment, and additional written information provided	Sham diet	88% (self‐reported)
Denmark, RCT, crossover [39]	*n* = 19 (89%) Patients with UC in remission meeting the ROME IV criteria for IBS (*n* = 19)	2	Dietary advice (duration not stated) from a dietitian with additional lists of foods to be self‐supplied	Typical Danish Diet	100% (self‐reported)
Italy, feeding RCT, parallel [36]	*n* = 55 (56%) Patients CD (*n* = 35) or UC (*n* = 20) in remission or with mild disease activity	6	Dietary advice from a dietitian during a 30–45 min appointment. Meal plan with three menu options provided at each main meal and additional meal preparation written information provided	Standard diet containing a FODMAP content typical of the Italian diet	Not reported
Australia Feeding RCT, crossover [38]	*n* = 9 (67%) Patients with quiescent CD (*n *= 9)	9	Almost all food provided as frozen complete meals, and additional food lists provided to enable purchase of additional low FODMAP foods	Typical Australian diet	89% (self‐reported)

Abbreviations: CD, Crohn's disease; GI, gastrointestinal; IBD, inflammatory bowel disease; IBS, irritable bowel syndrome; NA, not applicable; UC, ulcerative colitis;.

With regard to the delivery of the interventions, one trial was a feeding trial [38], and four used dietary counselling either from an expert dietitian [36, 37, 39] or a nutritionist [40]. With regard to control or comparator diets, one trial used a habitual diet [40], one used a standard diet containing a FODMAP content typical of the Italian diet [36], one used a ‘typical Danish’ diet [39], one used a sham diet [37], and the feeding trial used a ‘typical Australian’ diet [38] (Table 1). Of the five included RCTs, only three reported data on adherence to the intervention. All three studies reported high adherence, with 88%–100% of participants complying with the low FODMAP diet [37, 38, 39].

The two studies reporting qualitative data included a total of 30 participants, who were predominantly female (≥ 75%) with a mean age of 34.5–40.25 years (Table 2). Both studies reported on the lived experience of patients with IBS implementing a low FODMAP diet [19, 41].

**Table 2:** Characteristics of included qualitative studies of patient experiences of a low FODMAP diet.

Country, design	Population, healthcare setting	*N* (% female) Mean age (years)	Mode of delivery for FODMAP education	Phenomenon of interest	Method	Analysis	Main themes
New Zealand, Observational, mixed‐methods [41]	IBS, community	22 (77%) 34.5 years	Initial 90‐min group education session from a dietitian 60 min follow‐up session for patients with symptoms improved by ≥ 50% after 9 weeks Use of PowerPoint and written information provided	Explore the experiences, feasibility and effectiveness of a dietitian‐led low FODMAP diet group education programme.	Semi‐structured interviews	Thematic inductive approach	A new way of eating Replacement of foods Eating out Social support Resources Label reading.
UK, qualitative [19]	IBS, primary and secondary	8 (75%) 40.3 years	Basic printed low FODMAP resources provided by GPs and GEs	Lived experience of how people with IBS use and apply low FODMAP diet information provided by GPs and GEs to self‐manage symptoms.	Semi‐structured interviews	Interpretive phenomenological analysis, described as an iterative four‐step process	Validity of information Burden of use of information Effects on food‐related quality of life.

Abbreviations: GE, gastroenterologist; GP, general practitioner; IBS, irritable bowel syndrome.

### 6.2 Quality Assessment

#### 6.2.1 RCTs

**Figure 2:**
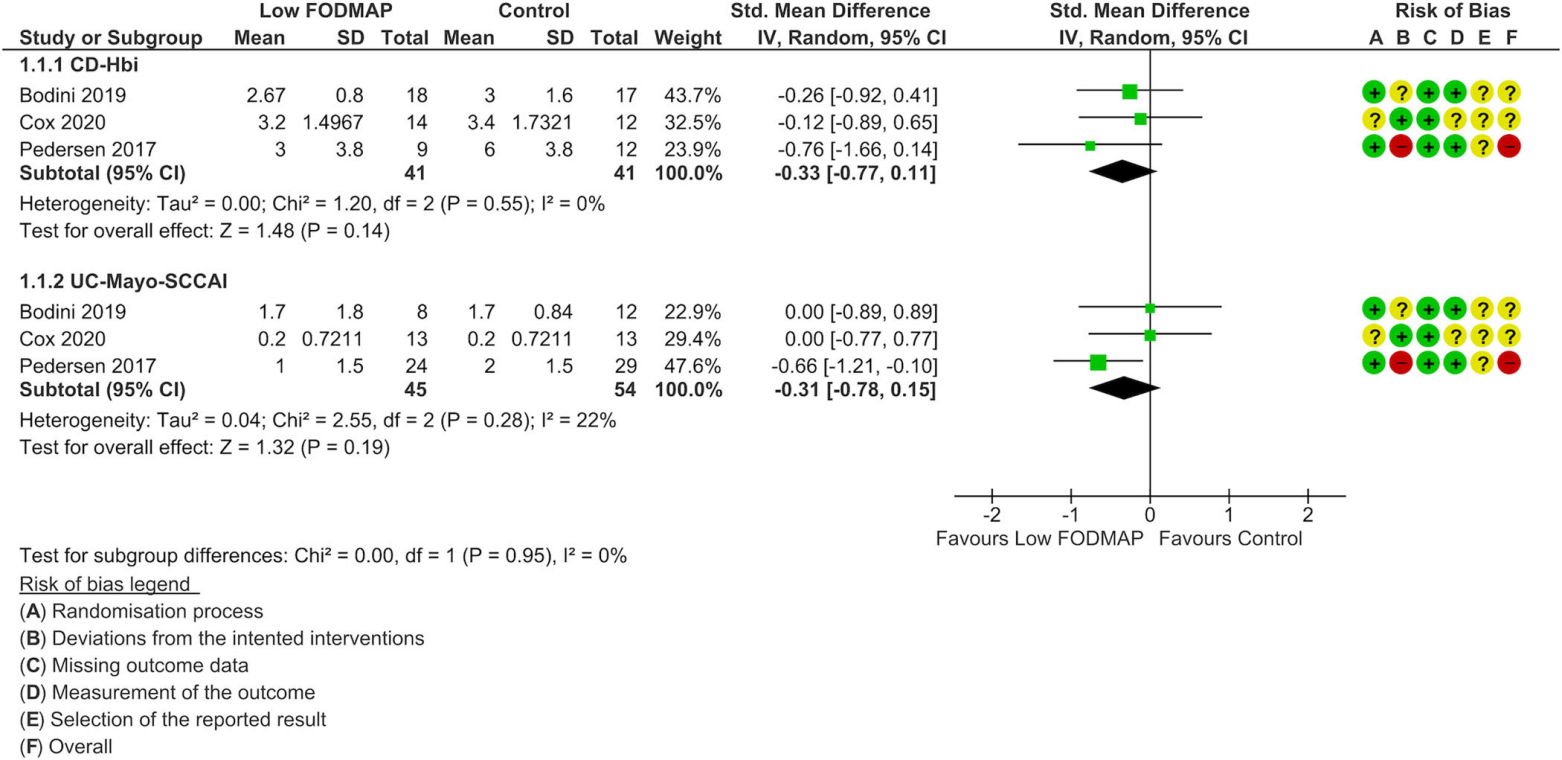
Forest plot for disease activity scores at the end of intervention.

**Figure 3:**
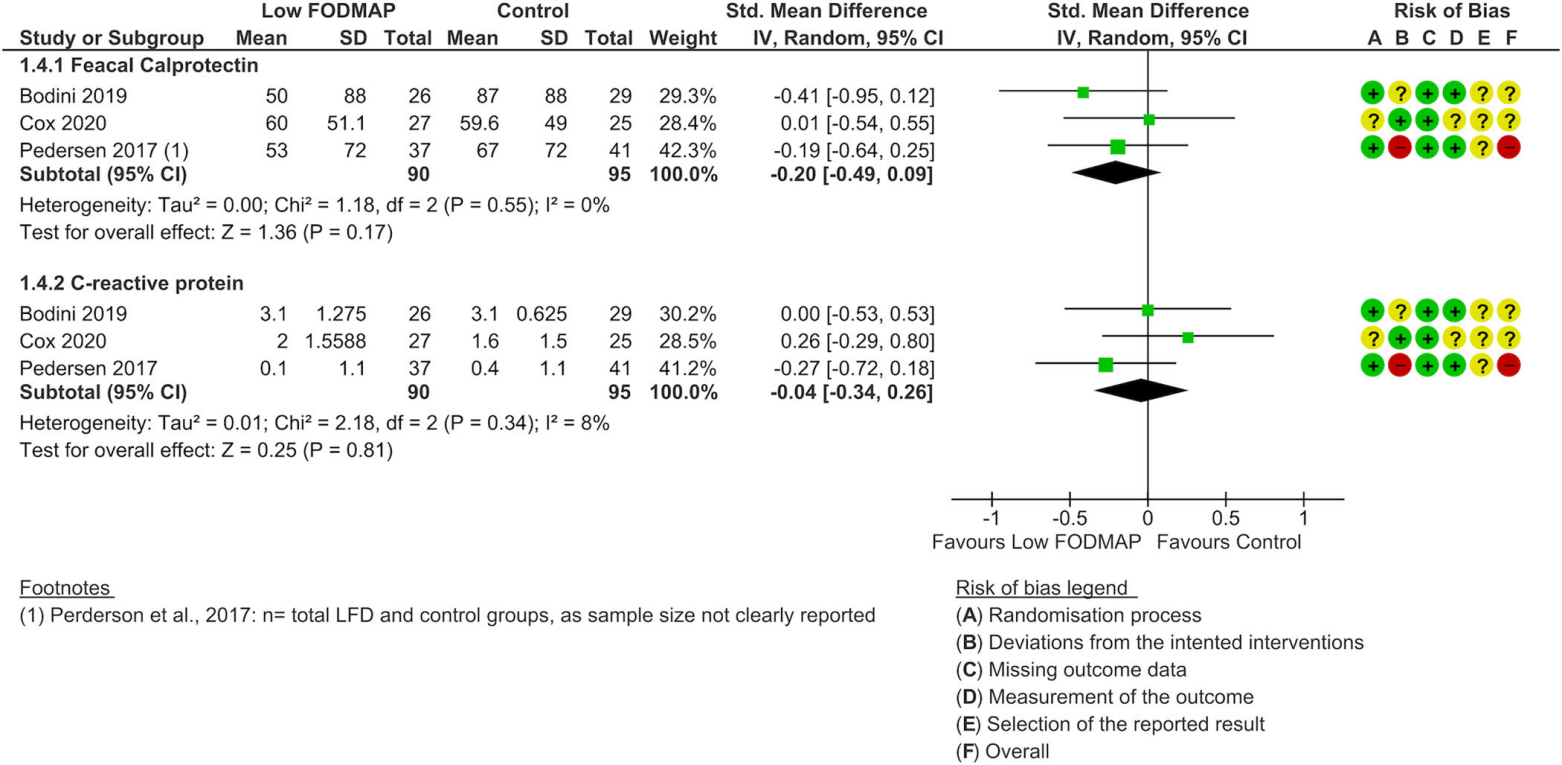
Forest plot for inflammatory markers scores at the end of intervention.

**Figure 4:**
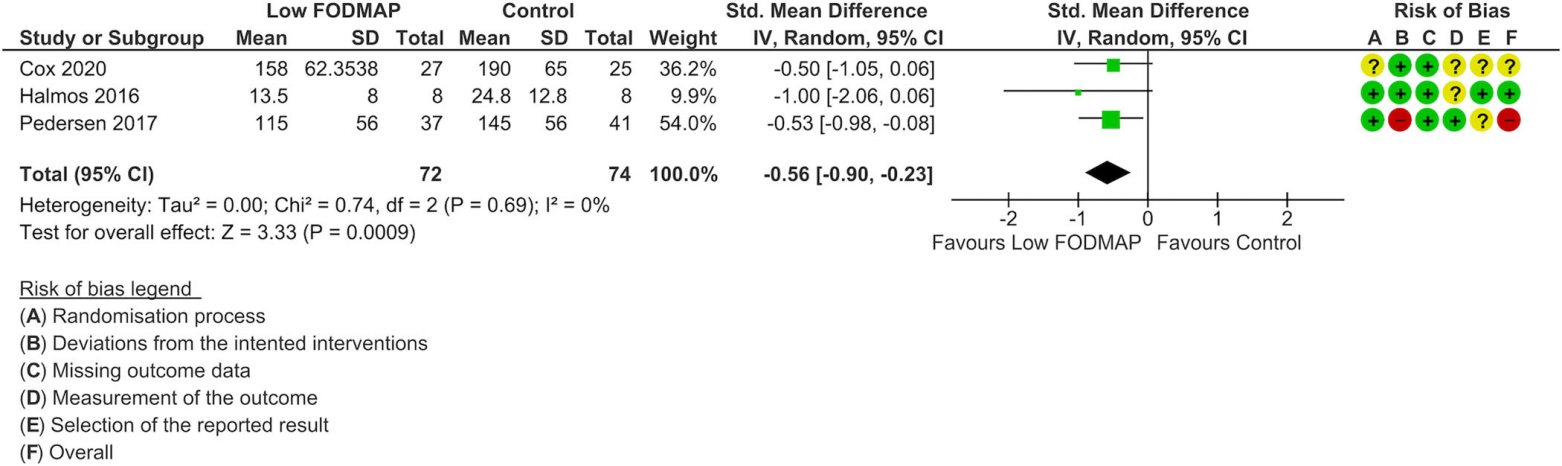
Forest plot for global gastrointestinal symptoms severity score at the end of intervention.

**Figure 5:**
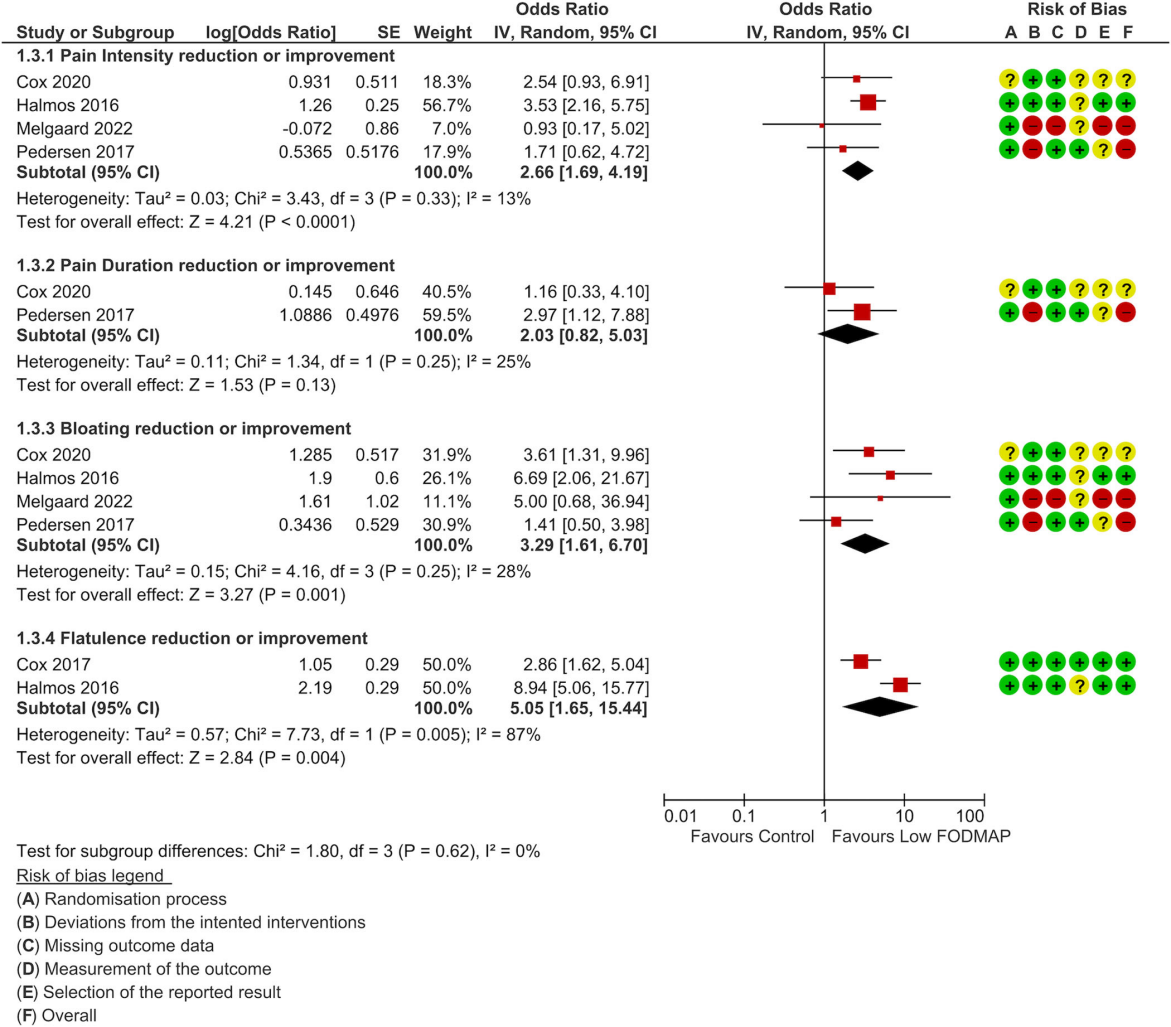
Forest plot for improvement of individual symptom scores.

**Figure 6:**
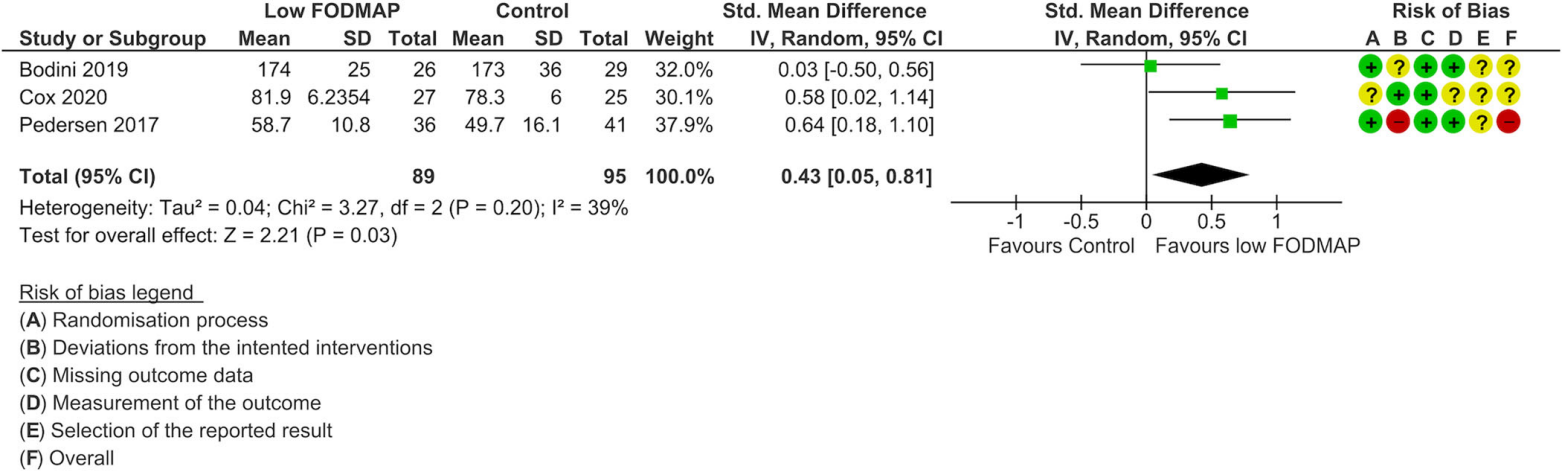
Forest plot for quality of life scores at the end of intervention.

None of the included RCTs were deemed as low risk of bias across all outcomes of interest. For disease activity outcome, one study was rated as high risk of bias [40] and two had some concerns [36, 37] (Figure 2). For inflammatory marker outcomes, one study was rated as low risk of bias [37], one had some concerns [36] and one was high risk [40] (Figure 3). For IBS‐symptom severity [37, 38, 40], abdominal pain intensity [37, 38, 39, 40], duration [37, 40], bloating [37, 38, 39, 40], flatulence [37, 38] and quality of life outcomes [36, 37, 40], all studies were classified as high risk of bias (Figures 4, 5, 6) [36, 37, 38, 39, 40].

#### 6.2.2 Qualitative Data Reporting Patient Experiences of the Low FODMAP Diet

One study met all Mixed Methods Appraisal Tool (MMAT) criteria for qualitative research [19] whilst the other study lacked clarity on the qualitative approach, and appropriateness and justification for the integration of qualitative and quantitative components when assessed against the mixed‐methods criteria (Supporting Information S1: 2) [41].

### 6.3 Meta‐Analysis

Summary of findings for outcomes reported in ≥ 2 RCTs and included in the meta‐analysis are reported in Table 3.

**Table 3:** Summary of findings for outcomes reported in ≥ 2 randomised controlled trials and included in the meta‐analysis.

		Results			Heterogeneity	
Outcomes	Number of participants (studies)	Effect size SMD or OR	95% CI	*p* value	*I* ^2^ (%)	Certainty of the evidence (GRADE)
Disease activity						
Crohn's	82 (3 RCTs) [36, 37, 40]	SMD: −0.33	−0.77 to 0.11	0.14	0%	⊕⊖⊖⊖ Very Low
Ulcerative colitis	99 (3 RCTs) [36, 37, 40]	SMD: −0.31	−0.78 to 0.15	0.19	22%	⊕⊖⊖⊖ Very Low
Inflammatory markers						
Faecal calprotectin	90 (3 RCTs) [36, 37, 40]	SMD: −0.20	−0.49 to 0.09	0.17	0%	⊕⊖⊖⊖ Very Low
C‐reactive protein	95 (3 RCTs) [36, 37, 40]	SMD: −0.04	−0.34 to 0.26	0.81	8%	⊕⊖⊖⊖ Very Low
Global gastrointestinal symptom severity	146 (3 RCTs) [37, 38, 40]	SMD: −0.56	−0.90 to −0.23	0.0009*	0%	⊕⊕⊖⊖ Low
Individual symptoms						
Abdominal pain intensity	121 (4 RCTs) [37, 38, 39, 40]	OR: 2.66	1.69– 4.19	< 0.0001*	13%	⊕⊕⊖⊖ Low
Abdominal pain duration	95 (2 RCTs) [37, 40]	OR: 2.03	0.82 to 5.03	0.13	25%	⊕⊕⊖⊖ Low
Abdominal bloating or distension severity	121 (4 RCTs) [37, 38, 39, 40]	OR: 3.29	1.61 to 6.70	0.001*	28%	⊕⊕⊖⊖ Low
Flatulence	68 (2 RCTs) [37, 38]	OR: 5.05	1.65– 15.44	0.004*	87%	⊕⊕⊖⊖ Low
Quality of life	184 (3 RCTs) [36, 37, 40]	SMD: 0.43	0.05– 0.81	0.03*	39%	⊕⊕⊖⊖ Low

Abbreviations: CI: confidence interval; GRADE: Grading of Recommendations Assessment, Development and Evaluation; OR: odds ratio; SMD: standardised mean difference.

*I* ^2^, with significant heterogeneity defined as *I* ^2^ ≥ 50%.

* Statistically significant (*p* < 0.05).

### 6.4 Disease Activity

Three RCTs (*n* = 181) investigated the effect of a low FODMAP diet on IBD disease activity. For those including patients with Crohn's disease, all used the Harvey–Bradshaw Index (HBI) [36, 37, 40]. For the three studies including patients with ulcerative colitis, two used the Mayo score [36, 37] and one used the Simple Clinical Colitis Activity Index (SCCAI) [40]. Overall, there was no difference in disease activity between a low FODMAP diet and controls at the end of the intervention in patients with Crohn's disease (SMD, −0.33; 95% CI −0.77 to 0.11; *p* = 0.14; *I* ^2^ = 0%; very low‐certainty evidence; Figure 2) or in patients with ulcerative colitis (SMD, −0.31; 95% CI −0.78 to 0.15; *p* = 0.19; *I* ^2^ = 22%*;* very low‐certainty evidence; Figure 2) [36, 37, 40].

### 6.5 Inflammatory Markers

Three RCTs (*n* = 90) reported the effect of a low FODMAP diet on faecal calprotectin and three RCTs (*n* = 95) reported the effect on CRP [36, 37, 40]. Overall, there were no significant difference between low FODMAP diet and controls at the end of intervention for faecal calprotectin (SMD: −0.20; 95% CI −0.49 to 0.09; *p* = 0.17; *I* ^2^ = 0%; very low‐certainty evidence; Figure 3) [36, 37, 40] or CRP (SMD: −0.04; 95% CI −0.34 to 0.26; *p* = 0.81; *I* ^2^ = 8%; very low‐certainty evidence; Figure 3) [36, 37, 40].

### 6.6 Global Gastrointestinal Symptom Severity

Three RCTs (*n* = 146) examined the effect of a low FODMAP diet on the severity of GI symptoms [37, 38, 40]. Two RCTs used the IBS‐symptom severity scale (IBS_SSS) questionnaire [37, 40] and one used a 100 mm visual analogue scale for overall symptoms [38]. There was a lower total IBS symptom severity score in the low FODMAP group at the end of intervention compared with controls (SMD: −0.56; 95% CI −0.90 to −0.23; *p* = 0.0009; *I* ^2^ = 0%; low certainty evidence; Figure 4) [37, 38, 40].

### 6.7 Individual Symptoms

#### 6.7.1 Abdominal Pain Intensity and Duration

Four RCTs (*n* = 121) reported on the effect of a low FODMAP diet on abdominal pain severity [37, 38, 39, 40], and two RCTs (*n* = 95) reported on the effect of a low FODMAP diet on pain duration [37, 40]. Overall, a low FODMAP diet led to a reduction in abdominal pain intensity compared with the control group (OR: 2.66; 95% CI 1.69–4.19; *p* < 0.0001; *I* ^2^ = 13%; low‐certainty evidence; Figure 5), with no statistically significant difference in abdominal pain duration between the two groups (OR: 2.03; 95% CI 0.82–5.03; *p* = 0.13; *I* ^2^ = 25%; low‐certainty evidence; Figure 5) [37, 38, 39, 40].

#### 6.7.2 Abdominal Bloating or Distension Severity

Four RCTs (*n* = 121) reported on the effect of a low FODMAP diet on abdominal bloating or distension severity [37, 38, 39, 40]. Overall, a low FODMAP diet led to a reduction of bloating or distention severity compared with controls (OR: 3.29; 95% CI 1.61–6.70; *p* = 0.001; *I* ^2^ = 28%; low‐certainty evidence; Figure 5) [37, 38, 39, 40].

#### 6.7.3 Flatulence

Two RCTs (*n* = 68) reported on the effect of a low FODMAP diet on flatulence [37, 38]. A low FODMAP diet led to a reduction of flatulence compared with controls (OR: 5.05; 95% CI 1.65–15.44; *p* = 0.004; *I* ^2^ = 87%; low‐certainty evidence; Figure 5) [37, 38].

### 6.8 Quality of Life

Three RCTs (*n* = 184) reported on the effect of a low FODMAP diet on quality of life using the IBD‐Questionnaire [36, 37, 40]. The low FODMAP diet resulted in higher quality of life score at the end of intervention compared with controls (SMD: 0.43; 95% CI 0.05–0.81; *p* = 0.03; *I* ^2^ = 39%; low‐certainty evidence; Figure 6) [36, 37, 40]. There were no studies measuring food‐related quality of life.

### 6.9 Dietary Intake and Bodyweight

Two RCTs reported on intake of nutrients in the diet groups at end of trial (*n* = 61) [37, 38]. One RCT used 7‐day food records [37], while the other used 21‐day food records to measure dietary intake [38].

In one trial, energy intake was significantly lower in the low FODMAP group compared with sham at the end of trial (1697 (47) vs. 1918 kcal/day (49), *p* = 0.002). The intake of protein and fat was lower in the low FODMAP group compared with sham (*p* = 0.008; *p* = 0.035, respectively). In addition, there were lower intakes of sugar, calcium, sodium, phosphorous and iodine in the low FODMAP diet group compared with sham at the end of the trial. However, there was no difference in the proportion of patients meeting the recommendations for macronutrients, micronutrients and fibre between the two groups [37] (Supporting Information S1: 3 **)**. In the other RCT, there was no significant difference in energy, or macronutrient intake between groups at the end of the trial [38] (Supporting Information S1: 3 **)**. None of the studies included reported changes in bodyweight.

### 6.10 Microbiome

Two RCTS measured microbiome composition [37, 38]. One used metagenomic sequencing to measure faecal microbiome composition and diversity [37]. There was no difference in gene count, species abundance or alpha or beta diversity between groups at the end of the trial [37]. In a targeted analysis, there was no difference between groups for relative abundance of total *Bifidobacteria*; however, there were some differences in specific *Bifidobacteria* species, such as *Bifidobacterium longum,* that was lower after a low FODMAP diet compared with sham diet controls (*p* = 0.005, *Q* = 0.017). In the targeted analysis, abundance of total *F. prausnitzii* species was also significantly lower after the low FODMAP diet group compared with sham at the end of the study (*p* = 0.038).

The crossover study used quantitative polymerase chain reaction to measure abundance of total bacteria and abundances of select groups (i.e., butyrate‐producing bacteria and mucus‐degrading bacteria) from 5‐day pooled faecal samples from seven participants [38]. Absolute and relative abundance of *Clostridum* Cluster XIVa and *Akkermancia muciniphila* were lower after the low FODMAP diet compared with controls, but there were no significant differences in absolute and relative abundance of *Lactobacillus* and *Bifidobacteria* spp. between groups.

### 6.11 Qualitative Reports of Experiences of Low FODMAP Diet

**Figure 7:**
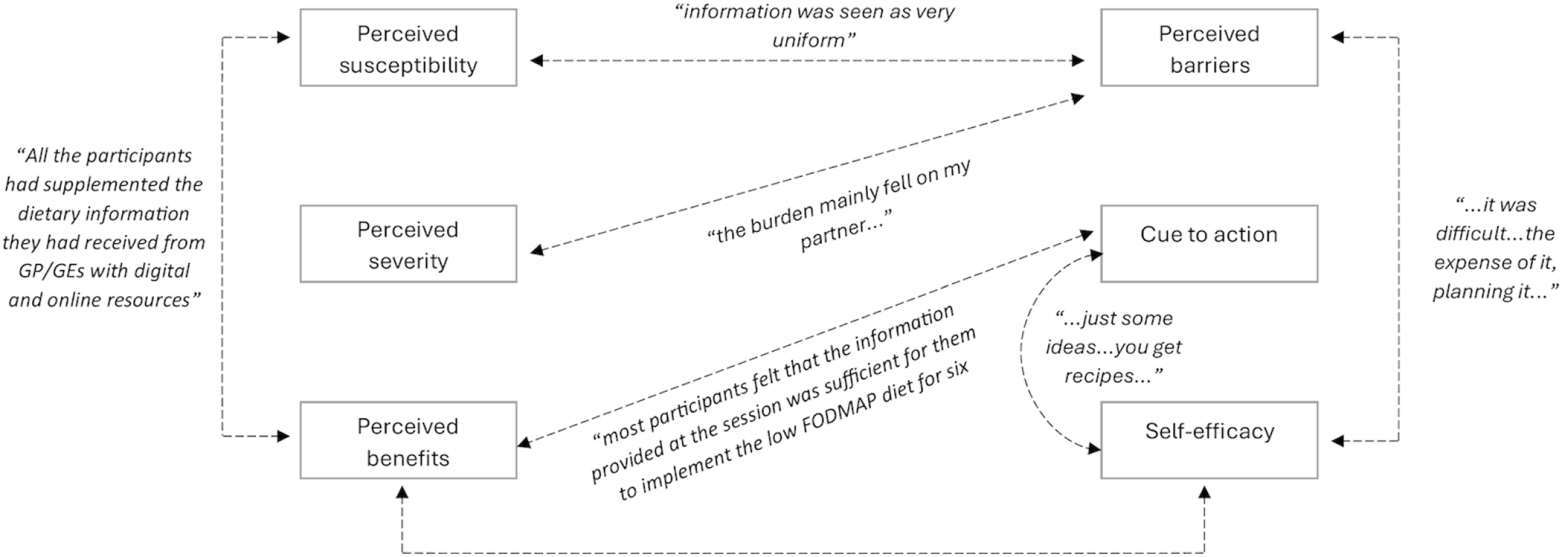
Interrelated domains of the Health Belief Model [34]. Oversimplified information from general practitioners (GPs) and gastroenterologists (GEs) may increase perceived susceptibility to negative effects of a restrictive diet, creating barriers to low FODMAP diet implementation. Perceived severity related to the impact on the individual's household and social impacts when implementing a low FODMAP diet. External cues, such as information sessions and meal ideas, were found to enhance knowledge and self‐efficacy, aiding individuals in successfully adopting dietary changes.

The two studies exploring the experiences of IBS patients implementing a low FODMAP diet revealed several challenges, mapped to all six domains of the Health Belief Model [19, 35, 41]. Our analysis identified all six domains to be represented and interrelated, highlighting potential enablers and barriers to implementation experienced by patients (Figure 7). Social difficulties (e.g., eating out) and the need for more supporting resources (to reduce the diet's impact on other members of the household or to manage social events) were reported across both studies [19, 41]. Patients suggested that tailored recipes or menu ideas would be beneficial when implementing a low FODMAP diet [19, 41]. Many found interpreting dietary advice and meal planning burdensome, turning to online resources even though they were unsure of their reliability [19]. A mobile app [43] was highlighted as helpful, while generic advice from health professionals was seen as inadequate [41].

## 7 Discussion

This review investigated the impact of a low FODMAP diet on IBD disease activity, inflammatory markers, GI symptoms, quality of life, diet quality and microbiome in adults living with IBD, and also evaluated patient experiences of implementing the low FODMAP diet. We found evidence that a low FODMAP diet leads to lower global symptom severity scores, abdominal pain severity, bloating and flatulence compared with control diets in IBD, and this was also associated with better quality of life. This aligns with the evidence for a low FODMAP diet on GI function symptoms and quality of life in IBS [8, 44, 45], and suggests that a low FODMAP diet may provide similar benefits in patients with IBD.

Whilst we found evidence of an effect on GI symptoms, we identified no effect on IBD disease activity or inflammatory markers [36, 37, 40]. The lack of benefit may be a function of the small number of included studies, most of which involved participants in remission [36, 37, 38, 39, 40], with only two studies including participants with mild to moderate disease activity [36, 40]. Conversely, the lack of effect could be a true result, given that there is currently no reliable mechanism for a low FODMAP diet to modulate inflammatory response in IBD. In fact, preclinical studies suggest that galacto‐oligosaccharide and fructan supplementation may exert anti‐inflammatory effects in the gut [46], which contrasts with the approach of restriction of these carbohydrates in the low FODMAP diet. Other data suggest fructans may only have the capacity to modulate disease activity if the gut microbiota can sufficiently ferment the fructans, and in contrast, a pro‐inflammatory effect was observed in response to fructan supplementation in a subset of IBD patients with active disease [47]. The current uncertainty about the effects of fructans and other fermentable carbohydrates on inflammatory status, and the evidence that the low FODMAP diet may lead to a lower abundance of immune‐regulatory *Bifidobacteria* [20, 37, 38, 48, 49], underscores the importance of the reintroduction and personalisation phases which aim to avoid unnecessarily prolonged dietary restriction and individualise the diet for patients in the long term. There does not appear to be an anti‐*bifidogenic* effect of the low FODMAP diet in IBD, as reported by So et al. [20]; however, we only synthesised data from two studies, one of which was very small, that used different microbial assessment approaches. It is essential to emphasise that while the diet can alleviate functional GI symptoms, our results do not show that it addresses the underlying inflammatory process and should, therefore, be considered a symptom management strategy rather than a treatment for IBD.

We further sought to understand the experiences of individuals when implementing a low FODMAP diet and found surprisingly only two studies in IBS (and none in IBD) that focused on patient experiences. This is despite wide acknowledgement of the intervention's complexity and the resulting need for regular patient support [23]. Both studies explored experiences of the diet with individuals with IBS, rather than IBD, reflecting the origins of the intervention [50]. We integrated and mapped the categorised qualitative data to the six domains of the Health Belief Model [35], a well‐established behaviour change model that has been widely used to describe key predictors of health behaviours by individuals. Although the quantity of literature was limited, the reported experiences of IBS patients provide important information for health professionals recommending a low FODMAP diet to patients.

Our synthesis of patients' perceptions provides important findings for practice. Despite the full low FODMAP intervention involving phases of restriction, reintroduction, and personalisation and delivery by a dietitian [22, 43], it is clear from the qualitative studies included in this review that patients with IBS are being instructed in the restriction phase through over‐simplistic or piecemeal education by the wider health professional team [19]. This may lead to patients feeling unsupported, burdened by interpreting dietary information and sourcing additional information from potentially unreliable sources. Insufficient support from health professionals has the potential to lead to nutritionally inadequate and unnecessarily restrictive diets longer term, if the follow‐up for the reintroduction phase is missed [23]. Provision of meal suggestions and recipes, identified as cues to action, may be more helpful than a list of ingredients to exclude, and would go some way to reducing patient challenges encountered when implementing a low FODMAP diet [35]. When contemplating a low FODMAP diet for IBS‐type symptoms in IBD, a multidisciplinary team that includes a dietitian will provide support for all phases of the intervention, and ensure that dietary restrictions are considered along with overarching lifestyle and nutritional needs [50].

Future studies that specifically explore the experiences of patients with IBD are critical to better understand the needs of this group. This is important for a number of reasons. First, there is a wide evidence base for the role of diet in IBD. For example, therapeutic diet intervention can treat inflammation in active IBD can control existing complications, and may be used to prevent disease development [51]. Therefore, expectations of some patients with IBD of the potential benefit of diet for their disease may be very different from people with IBS who have had limited experience with diet playing a central role in their care. Second, patients with IBD may experience a range of other nutrition‐related concerns that result from their IBD, such as iron‐deficiency anaemia or unintentional weight loss, and report inadequate intakes of a range of micronutrients compared with healthy controls [5]. These issues will mean the implementation of a low FODMAP diet may be different from people with IBS for whom the diet may require fewer modifications, which may also differentially impact on uptake, burden and acceptability. Finally, people with inactive IBD who experience functional GI symptoms experience worse food‐related quality of life compared with people with IBD without gut symptoms [5], and therefore this must also be considered in understanding impacts on patient experience.

Our review has some limitations. First, the review is limited by the evidence available and most included studies had a small sample size (e.g., one trial involved < 10 participants) [38] which tend to show more extreme treatment effects than larger studies [52], partly overcome by using SMD within the meta‐analyses. Second, due to the genesis of the low FODMAP diet as a treatment for IBS, rather than IBD, the included studies with qualitative data focused on IBS patients only with no studies specific to IBD. Our findings are relevant for identifying general support needs for patients when implementing the intervention, but there may be additional considerations for individuals with IBD.

## 8 Conclusion

This systematic review and meta‐analysis demonstrates that a low FODMAP diet may lead to lower global and individual IBS symptoms, and better quality of life compared with control diets in patients with IBD, but does not impact disease activity or inflammatory markers. Therefore, the low FODMAP diet should only be considered by clinicians for improving functional GI symptoms rather than a treatment for IBD. Qualitative studies specific to patients with IBD are needed to better support the safe and effective implementation of the low FODMAP diet in this group.

## Author Contributions

**Aubane Ville:** data curation (equal), formal analysis (equal), investigation (equal), methodology (equal), resources (equal), validation (equal), visualisation (equal), writing – original draft preparation (equal), writing – review and editing (equal), project administration (equal). **Rebecca McRae:** data curation (equal), formal analysis (equal), investigation (equal), methodology (equal), resources (equal), validation (equal), visualisation (equal), writing – original draft preparation (equal). **Jiayen Nomchong:** data curation (equal), investigation (equal), methodology (equal), resources (equal), validation (equal), writing – original draft preparation (equal). **Dianne P. Reidlinger:** data curation (supporting), methodology (supporting), writing – review and editing (equal), conceptualisation (supporting), project administration (lead), supervision (equal). **Alexandra R. Davidson:** data curation (supporting), writing – review and editing (equal), supervision (supporting). **Loai Albarqouni:** writing – review and editing (equal), conceptualisation (equal), project administration (supporting), supervision (equal). **Heidi M. Staudacher:** methodology (supporting), writing – review and editing (equal), conceptualisation (supporting), conceptualisation (supporting).

## Conflicts of Interest

The authors declare no conflicts of interest.

## Peer Review

The peer review history for this article is available at https://www.webofscience.com/api/gateway/wos/peer-review/10.1111/jhn.70106.

## Supporting information


**Supporting File 1:** Search string.

**Supporting File 2:** Mixed Methods Appraisal Tool.

**Supporting File 3:** Daily Dietary Intake.

## Data Availability

The data that support the findings of this study are available on request from the corresponding author. The data are not publicly available due to privacy or ethical restrictions.
