# Supplementary material for: Effects of a Low FODMAP Diet in Inflammatory Bowel Disease and Patient Experiences: A Mixed Methods Systematic Literature Review and Meta‐Analysis
Source: J Hum Nutr Diet. 2025 Aug 5;38(4):e70106. doi: 10.1111/jhn.70106 (PMC12326053; doi:10.1111/jhn.70106)
Supplement: Supplementary file 1 — Supporting File 1: Search string. [file JHN-38-0-s002.docx]

**Supplementary file 1**

Search strings for PubMed, Cochrane, Embase, CINAHL

PubMed

("Inflammatory Bowel Diseases"[Mesh] OR "Inflammatory Bowel Diseases"[tiab] OR "Inflammatory Bowel Disease"[tiab] OR IBD[tiab] OR "Irritable bowel disease"[tiab] OR "Crohn Disease"[Mesh] OR Crohn*[tiab] OR "CD"[tiab] OR "regional enteritis"[tiab] OR ileitis[tiab] OR "ileitis"[MeSH Terms] OR "UC"[tiab] OR “Ulcerative colitis”[Mesh] OR "Ulcerative Colitis"[tiab] OR Colitis[tiab] OR “granulomatous enteritis”[tiab] OR “granulomatous colitis”[tiab] OR “proctosigmoiditis”[tiab]  OR “rectocolitis”[tiab] OR “rectosigmoiditis”[tiab]  OR “ulcerative proctocolitis”[tiab] OR “hemorrhagic proctocolitis”[tiab] OR “haemorrhagic proctocolitis”[tiab] OR “proctitis”[tiab] OR IBS[tiab]  OR “irritable bowel syndrome”[Mesh] OR “irritable bowel syndrome”[tiab]) AND ("FODMAP Diet"[Mesh] OR FODMAP[tiab] OR FODMAPS[tiab] OR "Fermentable, poorly absorbed, short-chain carbohydrates"[tiab] OR "Fermentable oligosaccharides, disaccharides, monosaccharides and polyols"[tiab] OR “Diet, Carbohydrate-restricted" [Mesh] OR “Carbohydrate-Restricted”[tiab] OR prebiotics[tiab] OR prebiotic[tiab] OR "Prebiotics"[Mesh])

Cochrane

([mh "Inflammatory Bowel Diseases"] OR "Inflammatory Bowel Diseases":ti,ab OR "Inflammatory Bowel Disease":ti,ab OR IBD:ti,ab OR "Irritable bowel disease":ti,ab OR [mh "Crohn Disease"] OR Crohn*:ti,ab OR CD:ti,ab OR "regional enteritis":ti,ab OR ileitis:ti,ab OR [mh ileitis] OR UC:ti,ab OR [mh "Ulcerative colitis"] OR "Ulcerative Colitis":ti,ab OR Colitis:ti,ab OR "granulomatous enteritis":ti,ab OR "granulomatous colitis":ti,ab OR proctosigmoiditis:ti,ab OR rectocolitis:ti,ab OR rectosigmoiditis:ti,ab OR "ulcerative proctocolitis":ti,ab OR "hemorrhagic proctocolitis":ti,ab OR "haemorrhagic proctocolitis":ti,ab OR proctitis:ti,ab OR IBS:ti,ab OR [mh "irritable bowel syndrome"] OR "irritable bowel syndrome":ti,ab) AND ([mh "FODMAP Diet"] OR FODMAP:ti,ab OR FODMAPS:ti,ab OR "Fermentable, poorly absorbed, short-chain carbohydrates":ti,ab OR "Fermentable oligosaccharides, disaccharides, monosaccharides and polyols":ti,ab OR [mh "Diet, Carbohydrate-restricted"] OR Carbohydrate-Restricted:ti,ab OR prebiotics:ti,ab OR prebiotic:ti,ab OR [mh Prebiotics])

Embase

('Inflammatory Bowel Disease'/exp/mj OR 'Inflammatory Bowel Diseases':ti,ab OR 'Inflammatory Bowel Disease':ti,ab OR IBD:ti,ab OR 'Irritable bowel disease':ti,ab OR 'Crohn Disease'/exp/mj OR Crohn*:ti,ab OR CD:ti,ab OR 'regional enteritis':ti,ab OR ileitis:ti,ab OR ileitis/exp/mj OR UC:ti,ab OR 'Ulcerative colitis'/exp/mj OR 'Ulcerative Colitis':ti,ab OR Colitis:ti,ab OR 'granulomatous enteritis':ti,ab OR 'granulomatous colitis':ti,ab OR proctosigmoiditis:ti,ab OR rectocolitis:ti,ab OR rectosigmoiditis:ti,ab OR 'ulcerative proctocolitis':ti,ab OR 'hemorrhagic proctocolitis':ti,ab OR 'haemorrhagic proctocolitis':ti,ab OR proctitis:ti,ab OR IBS:ti,ab OR 'irritable colon'/exp/mj OR 'irritable bowel syndrome':ti,ab) AND ('FODMAP Diet'/exp/mj OR FODMAP:ti,ab OR FODMAPS:ti,ab OR 'Fermentable, poorly absorbed, short-chain carbohydrates':ti,ab OR 'Fermentable oligosaccharides, disaccharides, monosaccharides and polyols':ti,ab OR 'low carbohydrate diet'/exp/mj OR Carbohydrate-Restricted:ti,ab OR prebiotics:ti,ab OR prebiotic:ti,ab OR ‘prebiotic agent’/exp/mj)

CINAHL

((MH "Inflammatory Bowel Diseases+") OR (TI "Inflammatory Bowel Diseases" OR AB "Inflammatory Bowel Diseases") OR (TI "Inflammatory Bowel Disease" OR AB "Inflammatory Bowel Disease") OR (TI IBD OR AB IBD) OR (TI "Irritable bowel disease" OR AB "Irritable bowel disease") OR (MH "Crohn Disease+") OR (TI Crohn* OR AB Crohn*) OR (TI CD OR AB CD) OR (TI "regional enteritis" OR AB "regional enteritis") OR (TI ileitis OR AB ileitis) OR (MH ileitis+) OR (TI UC OR AB UC) OR (MH "Colitis, Ulcerative+") OR (TI "Ulcerative Colitis" OR AB "Ulcerative Colitis") OR (TI Colitis OR AB Colitis) OR (TI "granulomatous enteritis" OR AB "granulomatous enteritis") OR (TI "granulomatous colitis" OR AB "granulomatous colitis") OR (TI proctosigmoiditis OR AB proctosigmoiditis) OR (TI rectocolitis OR AB rectocolitis) OR (TI rectosigmoiditis OR AB rectosigmoiditis) OR (TI "ulcerative proctocolitis" OR AB "ulcerative proctocolitis") OR (TI "hemorrhagic proctocolitis" OR AB "hemorrhagic proctocolitis") OR (TI "haemorrhagic proctocolitis" OR AB "haemorrhagic proctocolitis") OR (TI proctitis OR AB proctitis) OR (TI IBS OR AB IBS) OR (MH "irritable bowel syndrome+") OR (TI "irritable bowel syndrome" OR AB "irritable bowel syndrome")) AND ((MH "low FODMAP Diet+") OR (TI FODMAP OR AB FODMAP) OR (TI FODMAPS OR AB FODMAPS) OR (TI "Fermentable, poorly absorbed, short-chain carbohydrates" OR AB "Fermentable, poorly absorbed, short-chain carbohydrates") OR (TI "Fermentable oligosaccharides, disaccharides, monosaccharides and polyols" OR AB "Fermentable oligosaccharides, disaccharides, monosaccharides and polyols") OR (MH "Diet, Low Carbohydrate+") OR (TI Carbohydrate-Restricted OR AB Carbohydrate-Restricted OR (TI prebiotics OR AB prebiotics) OR (TI prebiotic OR AB prebiotic) OR (MH Prebiotics+))
