# Supplementary material for: Effects of a Low FODMAP Diet in Inflammatory Bowel Disease and Patient Experiences: A Mixed Methods Systematic Literature Review and Meta‐Analysis
Source: J Hum Nutr Diet. 2025 Aug 5;38(4):e70106. doi: 10.1111/jhn.70106 (PMC12326053; doi:10.1111/jhn.70106)
Supplement: Supplementary file 2 — Supporting File 2: Mixed Methods Appraisal Tool. [file JHN-38-0-s003.docx]

| RefID | First author | Year | Citation | S1. Are there clear research questions? | S2. Do the collected data allow to address the research questions? | 1.1. Is the qualitative approach appropriate to answer the research question? | 1.2. Are the qualitative data collection methods adequate to address the research question? | 1.3. Are the findings adequately derived from the data? | 1.4. Is the interpretation of results sufficiently substantiated by data? | 1.5. Is there coherence between qualitative data sources, collection, analysis and interpretation? | 4.1. Is the sampling strategy relevant to address the research question? | 4.2. Is the sample representative of the target population? | 4.3. Are the measurements appropriate? | 4.4. Is the risk of nonresponse bias low? | 4.5. Is the statistical analysis appropriate to answer the research question? | 5.1. Is there an adequate rationale for using a mixed methods design to address the research question? | 5.2. Are the different components of the study effectively integrated to answer the research question? | 5.3. Are the outputs of the integration of qualitative and quantitative components adequately interpreted? | 5.4. Are divergences and inconsistencies between quantitative and qualitative results adequately addressed? | 5.5. Do the different components of the study adhere to the quality criteria of each tradition of the methods involved? |
| --- | --- | --- | --- | --- | --- | --- | --- | --- | --- | --- | --- | --- | --- | --- | --- | --- | --- | --- | --- | --- |
| 32994615 | Chan | 2020 | Chan, D., Skidmore, P., O’Brien, L., Watson, S., & Gearry, R. (2020). A feasibility study investigating the impact of a dietitian-led low in fermentable oligosaccharide, disaccharide, monosaccharide and polyols diet group education programme with irritable bowel syndrome. *New Zealand Medical Journal*, *133*(1522), 42–51. | Yes | Yes | Can't tell | Yes | Can't tell | No | Can't tell | Can't tell | Yes | No | No | Yes | No | No | No | Can't tell | No |
| 31212668 | Trott | 2019 | Trott, N., Aziz, I., Rej, A., & Sanders, D. S. (2019). How patients with ibs use low FODMAP dietary information provided by general practitioners and gastroenterologists: A qualitative study. *Nutrients*, *11*(6), 1313-. https://doi.org/10.3390/nu11061313 | Yes | Yes | Yes | Yes | Yes | Yes | Yes |  |  |  |  |  |  |  |  |  |  |
