# Supplementary material for: Effects of a Low FODMAP Diet in Inflammatory Bowel Disease and Patient Experiences: A Mixed Methods Systematic Literature Review and Meta‐Analysis
Source: J Hum Nutr Diet. 2025 Aug 5;38(4):e70106. doi: 10.1111/jhn.70106 (PMC12326053; doi:10.1111/jhn.70106)
Supplement: Supplementary file 3 — Supporting File 3: Daily Dietary Intake. [file JHN-38-0-s001.docx]

**Supplementary file 3**: Daily Dietary Intake

|  | Cox et al., (2020) | | | Halmos et al., (2016) | | |
| --- | --- | --- | --- | --- | --- | --- |
|  | Low FODMAP  (n=27) | Sham  (n=25) | *P-value* | Low FODMAP | Australian Diet | *P-value* |
| Energy Intake | 1697 (47) kcal/d | 1918 (49)  kcal/d | **0.002** | 8.17 (7.09-9.24)  MJ/d | 8.17 (7.37-8.97)  MJ/d | 0.979 |
| Protein (g/day) | 74 (2) | 83 (2) | **0.008** | 98.1 (83.7-113) | 96.1 (84.7-107) | 0.361 |
| Fat (g/d) | 68 (4) | 80 (4) | **0.035** | 74.4 (51.9-97.0) | 71.6 (49.4-93.8) | 0.337 |
| Sugar  (g/d) | 63 (4) | 76 (4) | **0.022** | 122 (106-139) | 120 (103-137) | 0.468 |

*Data presented as mean (SEM)

**Data presented as mean and 95% Confidence Interval (CI)
